# Glymphatic System Dysfunction and Sleep Disturbance May Contribute to the Pathogenesis and Progression of Parkinson’s Disease

**DOI:** 10.3390/ijms232112928

**Published:** 2022-10-26

**Authors:** Andie Scott-Massey, Matthew K. Boag, Annie Magnier, Dharah P. C. F. Bispo, Tien K. Khoo, Dean L. Pountney

**Affiliations:** 1 School of Pharmacy and Medical Sciences, Griffith University, Southport, QLD 4222, Australia; 2 School of Medicine and Dentistry, Griffith University, Southport, QLD 4222, Australia; 3 School of Medicine, University of Wollongong, Wollongong, NSW 2522, Australia

**Keywords:** sleep, Parkinson’s, glymphatic

## Abstract

Parkinson’s disease (PD) is a multisystem alpha-synucleinopathic neurodegenerative disease and the most prevalent neurodegenerative disorder after Alzheimer’s disease with a high incidence rate in the elderly population. PD is highly multifactorial in etiology and has complex and wide-ranging pathogenic mechanisms. Environmental exposures and genetic predisposition are prominent risk factors. However, current evidence suggests that an intimate link may exist between the risk factor of sleep disturbance and PD pathogenesis. PD is characterized by the pathological hallmarks of alpha-synuclein aggregations and dopaminergic neuron degeneration in the substantia nigra. The loss of dopamine-producing neurons results in both motor and non-motor symptoms, most commonly, bradykinesia, tremor, rigidity, psychiatric disorders, sleep disorders and gastrointestinal problems. Factors that may exacerbate alpha-synuclein accumulation and dopamine neuron loss include neuroinflammation and glymphatic system impairment. Extracellular alpha-synuclein can induce an inflammatory response which can lead to neural cell death and inhibition of neurogenesis. The glymphatic system functions most optimally to remove extracellular brain solutes during sleep and therefore sleep disruption may be a crucial progression factor as well as a risk factor. This literature review interprets and analyses data from experimental and epidemiological studies to determine the recent advances in establishing a relationship between glymphatic system dysfunction, sleep disturbance, and PD pathogenesis and progression. This review addresses current limitations surrounding the ability to affirm a causal link between improved glymphatic clearance by increased sleep quality in PD prevention and management. Furthermore, this review proposes potential therapeutic approaches that could utilize the protective mechanism of sleep, to promote glymphatic clearance that therefore may reduce disease progression as well as symptom severity in PD patients.

## 1. Introduction

Parkinson’s disease (PD) is one of the world’s most rapidly developing neurodegenerative diseases, with prevalence projected to double to twelve million by 2040, after an increase to six million between 1990 and 2015 [1]. PD incidence increases from five to ten-fold between the ages of 60 and 90 [2]. PD contributed a total economic burden of $51.9 billion in the United States in 2017 [3].

The results of the meta-analysis by Noyce and colleages found that the following risk factors have a positive association with PD pathogenesis: family history, constipation, mood disorders, pesticides, head injury, rural living, beta-blockers, farming or agriculture, well water and depression [4]. Furthermore, recent PD review articles have highlighted emerging evidence, from epidemiological studies, animal studies and Alzheimer’s disease research, supporting that sleep disturbance may be a significant risk factor in PD pathogenesis [5,6].

PD targets the extrapyramidal system in the brain but can progress to having wide-ranging effects on both the central and peripheral nervous systems. PD pathophysiology is predominantly characterised by the progressive dopaminergic neurodegeneration in the substantia nigra (SN) in the midbrain which is responsible for motor control, cognitive functions, and limbic activity [7,8]. Therefore, the loss of the dopaminergic neurons in this region results in the hindrance of these functions, resulting in the cardinal motor and non-motor symptoms of PD. Prominent motor symptoms include bradykinesia, tremor, rigidity, postural instability, dystonia and hypomimia. Significant non-motor symptoms include depression, anxiety, sleep disorders, gastrointestinal problems, pain, fatigue, urinary problems and poor memory [9]. Barone and colleagues studied the prevalence of these non-motor symptoms using a multicentre survey and found that sleep disturbance after psychiatric symptoms was the most frequently reported non-motor symptom among the 1072 PD patients enrolled in the study [10].

**Figure 1:**
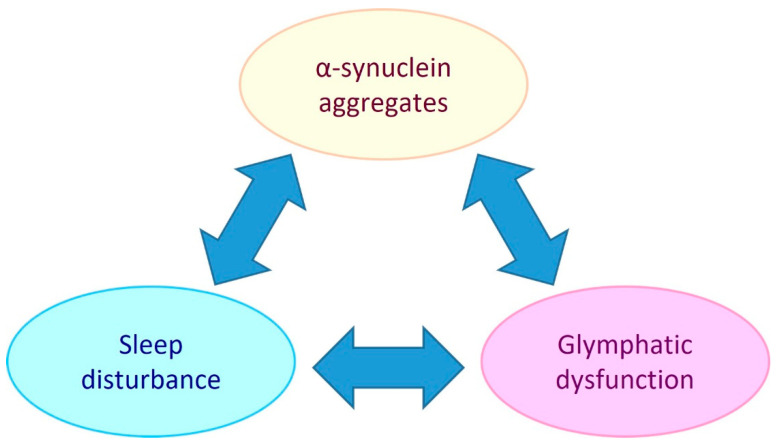
**Potential bidirectional relationships between alpha-synuclein aggregate pathology, the glymphatic system and sleep.** Sleep disturbance has been shown to be both a risk factor and chronic symptom of PD. Glymphatic dysfunction may contribute to PD by allowing extracellular accumulation of alpha-synuclein and other solutes. PD development may impair the glymphatic system by causing AQP4 depolarisation and reduced sleep. Glymphatic function has been found to be intimately dependent on sleep, so sleep disturbance could be both a symptom of PD and a causative element in PD pathogenesis by reducing glymphatic clearance.

PD is referred to as a synucleinopathy, which is a term that encompasses neurodegenerative disorders that have pathogenic lesions comprised of insoluble aggregations of the presynaptic protein, alpha-synuclein [11]. The pathogenic mechanisms that cause and facilitate the accumulation of alpha-synuclein in PD include mitochondrial dysfunction, oxidative stress, neuroinflammation, and glymphatic system impairment [12,13,14]. As there is an intimate link between sleep and the glymphatic system (extracellular solute clearance system of the brain) this suggests that bidirectional relationships exist between alpha-synuclein aggregate pathology, glymphaptic system dysfunction and the quality and/or duration of sleep (see Figure 1).

Idiopathic PD is the most prevalent form of Parkinsonism. However, there are other forms of parkinsonian disorders that include atypical parkinsonian disorders, which is a term used to distinguish neurodegenerative disorders which have similar signs and symptoms as PD but differ slightly in pathophysiology. The atypical parkinsonism syndromes include synucleinopathies; Multiple System Atrophy and Dementia with Lewy bodies, and tauopathies; Progressive Supranuclear Palsy and Corticobasal Degeneration [15]. Levin and colleagues found correlations between the synucleinopathies and rapid eye movement (REM) behaviour disorder (RBD) as well as impaired sleep [15]. The study did not detail a correlation between the tauopathies and sleep, however current studies affirm that there is an established link between sleep and Alzheimer’s disease which is a tauopathy [16]. Furthermore, all forms of parkinsonism involve some extent of dopaminergic denervation in certain regions of the brain. Since atypical parkinsonism disorders have similar clinical and pathological features as idiopathic PD, this review will focus on idiopathic PD to gain therapeutic insights that may be relevant for all forms of the disease.

Thus, this review hypothesises that glymphatic dysfunction and sleep disturbance are intimately linked to inducing PD pathology. This review aims to provide insight into the research question; to what extent does glymphatic system dysfunction and sleep disturbance play a role in PD pathogenesis and progression?

## 2. Molecular Pathology

PD is characterised by pathogenic forms of the intraneural protein called alpha-synuclein. When alpha-synuclein accumulates, this stimulates an aggressive inflammatory response from glial cells which are primed to defend the brain from pathogenic threats. If neuroinflammation becomes uncontrolled, this can lead to dopaminergic neurodegeneration and therefore cause PD [2].

### 2.1. Role of Alpha-Synuclein

Under normal physiology, alpha-synuclein is an abundant protein in the brain found in neuronal presynaptic terminals and plays a role in regulating presynaptic signalling, the stability of the neural cell membrane and membrane trafficking operation. Alpha-synuclein becomes pathogenic when it misfolds, is overexpressed due to genetic mutations or when it adopts oligomeric conformations or produces amyloid fibrils [17]. A study by Wegrzynowicz and colleagues used a transgenic mouse line (MI2) which expressed human, aggregation-prone truncated 1–120 alpha-synuclein and control mice to investigate alpha-synuclein pathology in PD [18]. Using stereological counting of cells stained for tyrosine hydroxylase (TH), whereby TH+ cells are a marker for dopaminergic neurons, progressive TH+ cell loss was observed in the SN of M12 mice, increasing in severity from 9 to 20 months. Furthermore, a substantial age-related decline in motor capacity in the rotarod test was observed in MI2 mice, compared to controls. Alpha-synuclein can also become toxic through the formation of intraneuronal inclusions called Lewy bodies [17]. Although the exact pathogenicity of Lewy bodies is still unclear, an immunohistochemical study by Power and colleagues found that Lewy bodies can extract mitochondria from nearby neural cell bodies and that pathological alpha-synuclein may degrade the nucleus and alter DNA [19]. This causes ATP depletion, damaged DNA and compromised axonal trafficking, resulting in neuron death.

### 2.2. Neuroinflammation

Several studies implicate neuroinflammation as playing a pivotal role in both PD onset and progression [20,21,22,23,24,25,26]. Some of these studies also show that alpha-synuclein aggregates can induce aggressive inflammatory responses in the brain that are predominantly mediated by microglia and astroglia which can lead to dopaminergic neurodegeneration.

#### 2.2.1. Microglia

Microglia are phagocytic cells that form the innate immune system defence in the central nervous system (CNS) and have both neuroprotective and neurotoxic functions [27]. These cells circulate in the brain parenchyma to maintain homeostasis, secrete neurotrophic factors, degrade toxic proteins and regulate neuron health [12]. Microglia density is significantly higher in the SN compared to other brain regions, which likely contributes to its high susceptibility to inflammatory insults [25].

Alpha-synuclein aggregation is a powerful inflammatory stimulator of microglia, which are primed to phagocytose or degrade the abnormal protein build-up [27]. When chronically activated, microglia secrete proinflammatory chemicals such as cytokines to neurotoxic concentrations which damage neurons. Consequently, cellular damage induces further glial cell mediated inflammatory responses which leads to progressive dopaminergic neurodegeneration that disseminates throughout the brain [12]. A study by Harms and colleagues investigated the role of the microglial-secreted inflammatory response complex, major histocompatibility complex II (MHCII) in neuroinflammation and neurodegeneration using viral-induced alpha-synuclein overexpression in mice [22]. The study found that alpha-synuclein overexpression caused substantial MHCII secretion from microglia, while MHCII knock-out resulted in an anti-inflammatory effect and prevented dopaminergic neuron loss. When microglia were treated with aggregated alpha-synuclein, this led to a cascade of antigen, T-cell, and cytokine release. Recent studies [21,23,24] conducted similar experiments as Harms and colleagues and concur with the findings [22]. Evidently, as alpha-synuclein continues to aggregate and form toxic fibrils, the inflammatory response becomes more aggressive and results in more widespread neurodegeneration.

#### 2.2.2. Astroglia

The most abundant glial cell in the CNS, astroglia are located in synaptic regions of neurons and have cytoplasmic projections with end-feet that cover approximately 97% of all cerebral vasculature. Astroglia have a neuroprotective role in maintaining neuronal metabolite homeostasis and regulating the permeability between the cerebral vasculature, interstitial fluid (ISF) and cerebrospinal fluid (CSF). Astroglia facilitate permeability through membrane water channels called aquaporin-4 (AQP4) which are highly localised in astrocytic end feet [28].

The study by Trudler and colleagues, showed that alpha-synuclein oligomers cause astrocytes to release glutamate which induces an inflammatory cascade that leads to synaptic damage and neuronal loss [29]. Similarly, Chou and colleagues demonstrated that alpha-synuclein fibrils cause downregulation of astrocyte phagocytic function and increased neurotoxic activity [30]. AQP4 dysfunction has also been implicated in promoting neuroinflammation and the accumulation of alpha-synuclein.

### 2.3. Aquaporin-4

Aquaporin-4 (AQP4) channels facilitate the CSF-ISF exchange of fluid and extracellular solutes between the perivascular space of brain vasculature and the interstitium [31]. Zhang and colleagues investigated AQP4 deficiency in experimental PD in mice and established that AQP4 deficiency generates an inflammatory response involving reactive astrogliosis, microgliosis and cytokine up-regulation in the SN [26]. Other studies support a similar relationship, whereby, decreased AQP4 expression exacerbated alpha-synuclein deposition, leading to increased microglia and astroglia activity, as well as reactive astrogliosis and in a bidirectional manner aggravated AQP4 depolarisation [32,33]. These effects coincided with dopaminergic neuron loss caused by inflammatory response hindering interstitial clearance, neurogenesis and damaging neuronal integrity. Therefore, neuroinflammation plays a significant role in PD pathology and may be exacerbated by impaired interstitial clearance and AQP4 deficiency.

## 3. The Glymphatic System

**Figure 2:**
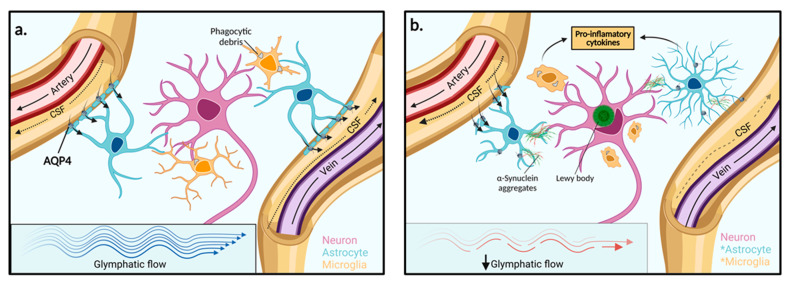
**Function of the glymphatic system in extracellular solute clearance.** (**a**) The subarachnoid space feeds CSF into the periarterial space of surface and penetrating cerebral arteries. The parenchymal ISF facilitates solute clearance via passage into the postcapillary vasculature. Flow is then directed back through the perivenous space towards the subarachnoid space via cerebral surface veins or cervical lymphatic drainage. In the interstitium, astrocytes line the vasculature with end-feet processes that express AQP4 channels in high concentration. (**b**) In PD, reduced glymphatic clearance may lead to the accumulation of extracellular α-synuclein aggregates and other solutes. Neighbouring neurons may then take up protein aggregates and facilitate the spread of Lewy body pathologies towards adjacent brain regions. The presence of aggregated α-synuclein within the extracellular space stimulates astrocyte and microglia activation to pro-inflammatory phenotypes and enhanced pro-inflammatory mediators. AQP4: Aquaporin 4, *: Activated cell type.

Named because of its high dependence on glial cells and its pseudo-lymphatic characteristic of clearing interstitial waste, the glymphatic system was first proposed by the study from Iliff and colleagues, who discovered a brain-wide fluid transport pathway in mice [34]. Before the glymphatic system, it was theorised that the CSF pathway was responsible for brain solute clearance. However, how solutes move from the brain parenchyma to the interstitium and then to the CSF to exit the brain was unknown [34]. Iliff and colleagues demonstrated, by injecting a fluorescent tracer into the cisterna magna (an opening in the subarachnoid space) of mice, that CSF interacts with the brain parenchyma by travelling along perivascular spaces that line penetrating arteries and veins (Figure 2) [34]. The route begins in the subarachnoid space where there is CSF influx into the perivascular spaces of cortical surface arteries and penetrating arterioles. The pathway then becomes surrounded by perivascular astrocytic end feet externally and the smooth muscle cells of the blood vessel internally. The pathway continues along parenchymal arterioles until it reaches the capillary beds in the brain. Here, fluid exchange occurs between the CSF in the pericapillary space and the interstitial space which collects solutes from the parenchyma. After the capillary beds, the ISF clearance is then facilitated along parenchymal venules where the pathway continues through perivenous pathways back towards the subarachnoid space. Some of the CSF-ISF mixture is then reabsorbed into the bloodstream by the arachnoid villi into the dural venous sinuses, which are surrounded by meningeal lymphatics and connect with the body’s conventional lymphatic system [34].

### 3.1. Physiology and Aquaporin-4 Water Channels

Lining the perivascular spaces are astrocytic end feet that are highly polarized with AQP4 water channels. AQP4 channels facilitate the movement of the CSF-ISF interchange whereby, CSF flows into the interstitium from the periarterial space and ISF, carrying all interstitial solutes, flows out of the brain parenchyma and into perivenous spaces to be cleared from the brain [34]. Other studies used CSF tracers injected into AQP4 knock-out (KO) mice and wild type (WT) mice to investigate glymphatic transport physiology in the parenchyma [34,35]. Both studies found that tracer movement into the parenchyma was significantly reduced in KO mice compared to WT mice.

Iliff and colleagues found that periarterial influx did not significantly reduce in KO mice due to the periarterial end-feet having significantly less AQP4 polarization compared to pericapillary and perivenous end-feet, leading to their conclusion that bulk flow is present in the periarterial influx pathway [34]. The study then supported that bulk flow drives interstitial solute clearance in the parenchyma after finding that interstitial-delivered tracers and amyloid-beta clearance was reduced by 70% and 55% respectively in KO mice [34]. Mestre and colleagues established that AQP4 channels play a significant role in CSF influx dynamics into the parenchyma and conducted a meta-analysis to examine the debate regarding the kinetics of fluid flow throughout the glymphatic system [35]. The study noted that earlier studies such as Iliff and colleagues did not have access to sufficient spatial resolution technology that could determine if bulk flow was universal to the entire interstitium [34]. The study highlighted several recent studies that support bulk flow and arterial pulsation in the periarterial space and support diffusion in the neuropil. Some of these studies that Mestre and colleagues appraised are discussed below [35].

### 3.2. Physical Forces Driving Fluid Movement

The fluid dynamics responsible for glymphatic flow are still heavily debated within the literature. Current theories of fluid movement mechanisms include diffusion, bulk flow, dispersion and arterial pulsatility. Some studies using tracer experiments in rodents supported that bulk flow (also known as ‘advection’ or ‘convection’) is a prominent mechanism of CSF and ISF movement, due to the rapid clearance of tracers along the perivasculature, irrespective of molecular size or concentration gradients [34,36,37].

Conversely, Faghih and Sharp found bulk flow to be implausible as it exceeded maximum perivascular resistance and pressure values that were determined using vascular tree models [38]. Jin and colleagues [39], who used spatial mathematical modelling based on primate brains and Smith and colleagues [40] who conducted rodent experiments also refuted the hypothesis of advective flow in the perivasculature and parenchyma and supported diffusion after observing size-dependent movement. Rey and Sarntinoranont also support diffusion to be a dominating mechanism, however, this study also investigates the role of arterial pulsatility and dispersion in providing rapid transport rates, as opposed to convection [41]. Other studies also support that cardiac, respiratory, and vasomotor pulsation patterns are associated with CSF flow dynamics [42,43].

Asgari and colleagues supported that pulsation driven bulk flow would be reliant on a valve mechanism which has not been found to date [44]. This study hypothesises that dispersion can provide fast solute transport and a greater distribution length of solutes in the brain. Other studies support that both diffusion and bulk flow are at play but to varying degrees, such that bulk flow may be more relevant in CSF transport or in large-solute transport where diffusion is too slow, whereas diffusion dominates in the parenchyma [45,46,47].

In light of the above literature, multiple mechanisms are likely at play in driving glymphatic fluid and transporting solutes in various regions in the brain. However, establishing these mechanisms holds particular relevance in neurodegenerative diseases due to their association with toxic solute clearance. Discrepancies in this area likely arise due to the difficulty in studying the glymphatic system and therefore a wide variety of methodical approaches have been used as human studies are not currently possible.

## 4. Glymphatic System Dysfunction in Parkinson’s Disease Pathology

Due to the complex physiology and crucial role the glymphatic system plays in brain health, emerging evidence has implicated glymphatic system dysfunction in neurological diseases, especially proteinopathies [31]. Brain health becomes increasingly vulnerable to protein-induced pathological insults as glymphatic system function inevitably declines with age. Consequently, mounting evidence has already established a causative link between Alzheimer’s disease and glymphatic impairment [48,49]. As a result of this underlying evidence, many studies now suggest that there is also an intimate link between glymphatic system dysfunction and PD pathogenesis [50,51,52,53,54,55,56].

Li and colleagues established that dilated or enlarged perivascular spaces (PVS) have been associated with over-abundance of extracellular proteins such as alpha-synuclein and tau as well as dopaminergic neurodegeneration in the SN in PD [53]. Park and colleagues found that enlarged PVS was associated with PD related pathological signatures such as white matter hyperintensity severity, higher levodopa-equivalent dose, hypertension, and lower mini-mental state examination [56]. Chung and colleagues also found that enlarged PVS-positive PD cases required higher doses of Levodopa, likely correlated with severely decreased dopamine transporter availability and a higher risk of freezing of gait [51]. Chen and colleagues also found presynaptic dopamine deficiency and motor dysfunction in PD patients with high severity of visible basal ganglia PVS on magnetic resonance imaging (MRI), in addition to advanced cognitive decline and increased levels of CSF toxic proteins [50]. The first two studies both sampled data from a specific hospital, whereas Chen and colleagues sampled from the Parkinson’s Progression Marker Initiative cohort [50]. Thus, generalisability could be lacking in these studies. However, because all three studies corroborate each other, this may lend support to their internal validity [51,56].

In conflict with the aforementioned studies, Donahue and colleagues investigated both global and regional changes in PVS and found that there was no significant difference between these regions between idiopathic PD and healthy controls [52]. However, this difference may be attributed to the difference in the regional examination as Donahue and colleagues did not study deep brain regions such as the basal ganglia [52]. Dopaminergic neurons in the SN form synaptic connections with neuron populations of the basal ganglia and thus PVS disturbance likely originates here before being detectable in global brain regions [8]. Differences could also be attributed to the small sample size, high variability and age-related comorbidities in the healthy control group.

The ALPS index is another indicator of a compromised glymphatic system and specifically, a low ALPS index indicates reduced diffusivity along the PVS. PD patients, especially those in the later PD stages, have a significantly lower APLS score than early stage PD, essential tremor patients and healthy patients [54,55]. These studies also found a low ALPS index correlated with enlarged PVS, poor scoring on mental state exams and periventricular white matter hypersensitivities. These studies are susceptible to regional bias due to the ALPS index as they are only calculated based on a specific brain region. Han and colleagues may correct this as this study used an alternate method to measure glymphatic flow in PD by measuring global blood-oxygen-level-dependent (gBOLD) signals coupled to CSF flow [57]. This study found an intimate relationship between global brain activity and CSF dynamics as well as with sleep. gBOLD signal strength significantly decreased in PD patients with cognitive dysfunction compared to controls or patients with only mild cognitive dysfunction. Strong signal strength was reported during sleep and also coupled CSF fluid movement [58]. Although McKnight and colleagues support that alpha-synuclein is implicated in dilated PVS seen in MRI, all three studies including Han and colleagues and Fultz and colleagues did not record regional values of alpha-synuclein to correlate index score or gBOLD signal with protein levels and so this relationship may still be unknown [55,57,58].

### 4.1. Compromised Aquaporin-4 Channels

AQP4 deficiency or altered polarity can induce neuroinflammation and also hinder glymphatic exchange in the parenchyma and these factors play a key role in PD pathology. Although alpha-synuclein is a dominant pathological hallmark, the relationship between AQP4 channels and alpha-synuclein has not been thoroughly examined in the literature. According to current experimental evidence there may be a bidirectional relationship between impaired glymphatic flow and AQP4 depolarisation, whereby reduced glymphatic flow through the brain can directly exacerbate alpha-synuclein pathology and also compromise AQP4 polarity [32]. However, reduced AQP4 expression also has the potential to further aggravate alpha-synuclein deposition as fluid exchange is hindered [33].

The experimental study, by Zou and colleages, blocked meningeal lymphatic drainage at the cervical lymph nodes in 24-week-old A53T and control mice models to investigate the correlation between glymphatic system dysfunction, AQP4 polarization and alpha-synuclein accumulation in PD pathology [32]. A more recent study conducted similar investigations, however, this study used AQP4 null mice (AQP4^+/−^) and control mice that were subjected to alpha-synuclein preformed fibril (PFF) injections and the effect on AQP4 expression and subsequently alpha-synuclein deposition in three brain regions were measured over six months [33].

The A53T-LDclns and AQP4^+/−^-PFFs mice models showed the highest AQP4 depolarisation, alpha-synuclein aggregation, dopaminergic neurodegeneration and inflammatory activity compared to the other mice models, indicating that impaired glymphatic flow is a powerful progression factor in a developing synucleinopathy [32,33]. However, when the pathological factors of glymphatic system impairment (by LDclns or AQP4^+/−^) and alpha-synuclein accumulation are working independently in the mice models, they still have pathogenic potential but precipitate effects of smaller magnitude. Furthermore, both studies tested the motor capability of the different mice models and found that motor coordination and balance was most significantly diminished in mice with a predisposition to excess alpha-synuclein (A53T or PFF) and with compromised glymphatic flow (either by LDclns or AQP4 deficiency), compared to the other mice models. Evidently, impaired glymphatic exchange by altered AQP4 polarity or direct blockage of fluid movement are prominent pathogenic and progression factors in PD pathology.

### 4.2. Sleep Disruption

**Figure 3:**
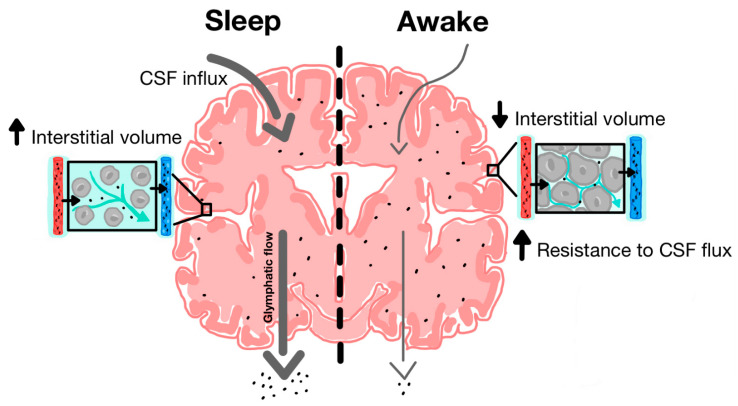
**Increased function of glymphatic clearance during sleep compared to wakefulness.** During sleep, there is increased CSF influx along periarterial spaces and increased ISF efflux along perivenous spaces. Cytomorphological changes are associated with increased interstitial volume. During wakefulness, there is decreased CSF influx and ISF efflux and most CSF is shunted out along lymphatic pathways due in part to decreased interstitial and perivascular volume [35].

The discovery of the glymphatic system and its role in clearing metabolic waste from the parenchyma has provided new insight into the neuroprotective role of sleep in facilitating glymphatic activity [14]. During natural sleep, there is a 60% increase in interstitial volume, contributing to a substantial increase in CSF-ISF exchange and extracellular solute removal [59]. Xie and colleagues tested CSF influx and amyloid-beta clearance in awake, anesthetized and sleeping mice, using in vivo photon imaging and ECoG power spectrum analysis to show the high power of slow-wave patterns that are indicative of deep sleep [59]. During a 30-min interval, there was a 95% reduction in periarterial and parenchymal tracer flow in awake mice as opposed to sleeping mice (Figure 3). Furthermore, intracortical injection of amyloid-beta was cleared two times faster in sleeping mice compared to awake mice.

Additionally, anaesthesia increased glymphatic influx and efflux, indicating that the sleep-wake state may be more responsible for determining the interstitial volume and glymphatic solute clearance efficacy compared to the circadian rhythm [59]. Conversely, Tuura and colleagues found, after using MRI and electroencephalogram analysis in rodents, there was an increase in water diffusivity during sleep which was positively correlated with the REM sleep stage and negatively correlated with the non-REM sleep stage [60]. Although CSF flow increases overnight were found to be unrelated to sleep and diffusivity measures, these findings may suggest interstitial clearance is optimal during REM sleep. Additionally, when adrenergic antagonists were administered or adrenergic signalling was inhibited, this also resulted in an increase in CSF tracer that was similar to observations in sleeping or anesthetised mice. This suggests that catecholamines may play a role in influencing interstitial volume [60]. This is further supported by the study, Hablitz and colleagues, which affirmed that increased cortical delta band power (electrical brain wave associated with deep sleep) and decreased heart rate correlated with improved glymphatic flow in the brain [61]. The strong correlation between quality deep sleep and glymphatic efficiency implicates sleep disruption as a potential pathogenic factor in the development of PD.

## 5. The Role of Sleep in Parkinson’s Disease

### 5.1. Lessons from Alzheimer’s Disease

The pathological link between sleep disruption and neurodegenerative diseases was first investigated in Alzheimer’s Disease (AD) [6]. AD typically begins in the hippocampus and entorhinal cortex and is characterised by the formation of amyloid plaques and tau tangles which form from amyloid-beta and tau accumulation, respectively, which can inhibit neurogenesis and induce neuronal death, leading to cognitive decline [62,63]. Iliff and colleagues studied the role of glymphatic dysfunction in disease by studying AD and amyloid-beta and observed that reduced clearance was also associated with increases in protein aggregation [34]. Sleep disruption is also a common early-stage symptom, therefore, mounting evidence has suggested that there may be a bidirectional relationship between sleep disturbance and AD pathology [16,63]. A cross-sectional study by Spira and colleagues found that shorter sleep duration and poor sleep quality was associated with increasing amyloid-beta burden [64]. Recent studies found, by systematic review and meta-analysis, that ~15% of AD cases were attributable to sleep disorders [65]. Shokri-Kojori and colleagues found that even after one night of sleep deprivation there were substantial increases in amyloid-beta accumulation in the hippocampus and thalamus in human subjects [66]. Due to the mounting evidence from AD, current research is focussed on sleep disruption as a risk factor in PD and similarities in the glymphatic dependence of extracellular protein clearance.

### 5.2. Circadian Rhythm and Clock Gene Dysfunction

Breen and colleagues investigated hormone levels and clock gene expression to investigate altered circadian rhythm in PD patients using polysomnography and serum analysis [67]. Controls experienced normal 24-hour oscillations in the expression of the clock gene, *Bmal1*, whereas the expression of *Bmal1* remained predominantly constant in PD patients. There was no observable difference between cases and controls in the other two studied clock genes, *Per2* and *RevErba*, however, in comparison to *Bmal1*, these clock genes do not play a significant role in circadian rhythm regulation [68]. PD patients also experienced reduced melatonin levels, elevated cortisol, as well as overall poor sleep quality characterised by increased sleep and REM latency, reduced sleep efficiency as well as a higher incidence of sleep disorders. Furthermore, Breen and colleagues proposed that dopamine may play a role in regulating *Bmal1* expression, hence the dopamine depletion in PD may have an indirect effect on circadian rhythm disruption [14]. In addition, the accompanying pathology of inflammation has also been implicated in clock gene and circadian rhythm disruption and a bidirectional relationship has also been explored, whereby circadian rhythm impairment is a contributor to abnormal inflammatory responses underlying PD pathogenesis [69,70]. 

Ono and colleagues found using in vitro studies that melatonin was able to inhibit alpha-synuclein assembly and destabilise fibrils and therefore prevent alpha-synuclein cytotoxicity and thus neurodegeneration [71]. Hence, reduced melatonin levels may have a direct effect on exacerbating alpha-synuclein pathogenicity. These findings reinforce that sleep is intimately connected with PD pathology. Thus, the possible association between PD and abnormal clock gene expression, circadian rhythm dysfunction and reduced REM sleep may explain the high prevalence of sleep disturbance complaints in PD patients. Furthermore, impaired sleep is strongly correlated with glymphatic dysfunction and therefore alpha-synuclein aggregation and dopaminergic neurodegeneration and thus, symptomatic sleep disruption may be a powerful progression factor in PD [14].

### 5.3. Sleep Disorders

Epidemiological studies have found sleep disturbance to be a strong risk factor in PD [72,73,74]. The most frequent prodromal non-motor symptom domains have been found to be GI tract (67.5%), sleep (52.6%), urinary tract (42.2%) and cardiovascular system (32.5%) [75]. Sohail and colleagues followed sleep fragmentation experienced by 269 adults (with no PD) until death and found that adults with sleep fragmentation had an increased number of Lewy bodies and SN neurodegeneration post-mortem [74]. Similarly, Lysen and colleagues conducted a nested cohort of 7725 adults and prospectively examined sleep quality [73]. The study found that poor sleep quality and decreased sleep duration was positively associated with increased PD risk in the next 6 years, however the strength in association weakened over longer follow up periods. Hence, the study also concluded that sleep disturbance is more likely a prodromal factor than a causal factor, however further research is needed [73]. 

Hsiao and colleagues examined the relationship between non-apnoea sleep disorders (NSD) and PD development by conducting a cohort study that recruited 91,273 adult patients with non-apnoea sleep disorders and no pre-existing PD from the Taiwan National Health Insurance Research Database between 2000 and 2003 [72]. A matched control cohort was selected, and all participants were followed until 2010 or death. Overall, NSD (sleep disturbance, insomnia or other circadian rhythm disruption, sleep-stage or arousal dysfunction or other sleep disturbances of non-organic origin) resulted in a significantly higher incidence rate and hazard ratio than controls. Chronic insomnia resulted in the highest incidence rate and hazard ratio among the NSD. Insomnia is the most common sleep disorder as it affects approximately one-third of the US population and has a severe impact on the elderly population and those with neurodegenerative disease [76,77,78]. Furthermore, the cumulative incidence of PD cases increased at a faster rate in the NSD cohort compared to controls over time. These results indicate that NSD may substantially increase the risk of developing PD, especially chronic insomnia which can potentially have a direct effect in reducing deep sleep quality.

Another prominent sleep disorder that is currently receiving significant attention in the literature as a potential prodromal biomarker to PD is REM behaviour disorder (RBD). This is because RBD disrupts the REM sleep stage and can lead to a vicious cycle of neuroinflammation, alpha-synuclein deposition and EPVS burden [79]. In a MRI study, RBD was associated with subtle changes in white matter integrity and grey matter volume in patients with early Parkinson’s disease [80]. Described as parasomnia, RBD patients experience a reduction in normal muscle atonia causing them to enact their dreams during REM sleep [81]. Si and colleagues investigated MRI-visible EPVS in idiopathic RBD and PD patients (with and without RBD) compared to controls [79]. iRBD patients had consistently higher EPVS burden in all studied brain regions (centrum semioval, basal ganglia, brainstem, and substantia nigra) compared to controls and all PD patients. Furthermore, PD and controls did not significantly differ in EPVS numbers which is similar to Donahue and colleagues but contradicts the several studies previously mentioned [52]. Furthermore, there was no significant difference in EPVS burden between PD-RBD and non-RBD PD patients. These results may be attributable to a possible compensatory role of EPVS in the early stages of PD compared to late stages as the average Hoehn & Yahr stage in participants was 2.0 [79]. Indeed, findings by Iranzo and colleagues indicated that in most patients diagnosed with IRBD this parasomnia represents the prodromal phase of a Lewy body disorder [82]. Lavault and colleagues showed that patients with clinical RBD were disabled earlier than patients without RBD, but there was no specific worsening in the RBD group with time [83]. A population-based study by Boot and colleagues found that probable rapid eye movement sleep behavior disorder increased risk for Parkinson’s disease [84]. Overall, the data suggests that RBD may be a stronger pathogenic factor rather than a progression factor in PD as EPVS burden did not increase in PD-RBD patients compared to just RBD patients. In support of these results, Shrestha and colleagues conducted a systematic review and concluded that RBD diagnosis is of high importance in early PD detection due to strong prodromal links [81]. The main finding by Schenck and colleagues was that 80.8% of iRBD diagnosed adult males aged at least 50 years of age were subsequently diagnosed with a parkinsonian or a dementia related disorder 5–29 years later [85]. Postuma and colleagues found an average phenoconversion rate of 6.3% per year from iRBD to neurodegenerative disease and 73.5% of iRBD patients converted after 12 years [86].

Depression is another potential prodromal factor of PD that also has strong associations with sleep disturbance. According to polysomnography psychiatric research by Riemann and colleagues, patients with depression exhibited a decline in delta power, disinhibition of REM sleep, decrease in REM latency and an increase in REM density and total REM sleep time [87]. As previously mentioned, a high delta power and REM sleep stage was positively correlated with glymphatic clearance. Therefore, there is a discrepancy in whether depression improves or hinders glymphatic function. However, delta power may be more influential in glymphatic optimality as according to Hablitz and colleagues, glymphatic function was most optimal during anesthesia which involves high delta power [61]. Hence, the glymphatic system may be more active during deep sleep (a stage in non-REM sleep) rather than REM sleep as this sleep stage experiences a decreased delta power. Despite the uncertainty surrounding links to the glymphatic system, depression has been correlated to PD as a risk factor. Wang and colleagues conducted a meta-analysis and found that there was a significant positive association between depression and PD risk [88]. Another study, investigated the relationship between PD and insomnia as well as other comorbidities and found that the prevalence of insomnia and depression among PD patients was 48% and 24% respectively [89]. Major depressive disorder and PD have also been linked by glymphatic system dysfunction [90]. Hence, disorders that affect sleep such as chronic insomnia, RBD and depression may play a principal role in PD pathogenesis and progression. Table 1 summarizes the current work indicating links between PD, sleep and glymphatic disturbance.

## 6. Limitations of the Current Research

There are limited primary source data on human neuropathology due to ethical constraints. Consequently, the majority of experimental studies used in this review are animal studies. Human and intervention studies, which would provide the highest quality of evidence, are lacking. Epidemiological studies overall were also lacking, esspecially intervention studies which are crucial in research being the only epidemiological study design that can establish causality. Of the epidemiological studies used, many were cohort or case–control studies, which although they have acceptable scientific rigor, are only able to establish an association.

Of the human studies, many utilised MRI imaging or ex vivo brain tissue due to the difficulty and unethical nature of conducting invasive experiments on the live human brain or using complex human brain surrogates [91]. Ex vivo brain tissue is obtained either from the deceased or from surgical procedures where it is removed to treat pathologies. Although this approach allows for morphological mapping, cellular extraction, and neuron manipulation, this method lacks sensory input and motor output generation and communication within the brain [92]. Conversely, MRI studies can achieve this depth of physiological investigation. However, MRI methods can lack the required sensitivity to detect changes on a cellular level [93].

Among the animal studies, many involved genetically engineered animals where certain genes have been knocked out (i.e., AQP4) or overexpressed (i.e., A53T) and other animals studies involved CSF tracers. Not all aspects of human neurological disease can be recapitulated in animal models due to the fundamental differences in neurodevelopment, genetics, pathology, and disease mechanisms [93]. For instance, the neurobiological importance of sleep for glymphatic function may not be as crucial in animals that do not perform higher-order functioning and may not rely on strict sleep-wake cycles.

The lack of human and intervention studies which would provide primary and high-quality data on relationships between sleep, glymphatic impairment and PD, have led to the difficulties in establishing a strong causal link in this review. Because the glymphatic system is still a recently described phenomenon and its relationship with PD and sleep is still actively being studied, many studies cited within this review did not provide definitive conclusions, rather the majority of studies suggested further research is needed.

## 7. Potential Therapeutic Approaches

If increased glymphatic flow and higher quality sleep are neuroprotective factors of PD, then this may give insight into future therapeutic approaches that could be implemented to reduce the risk and progression of PD. Firstly, deep brain stimulation has been found to significantly increase sleep quality and motor function in PD patients by increasing deep sleep and slow-wave activity. However, it is uncertain whether this technique normalises the sleep cycle as opposed to alleviating sleep disruption [94,95,96]. Another therapeutic technique, investigated by Pastukhov and colleagues is the administration of U-133, a chaperone inducer, which increased deep sleep and slow-wave activity in the preclinical PD stages of aged rats [97]. U-133 may hold promise as a PD prophylactic therapy in the older population or in slowing disease progression. Randomized control trials (RCTs) have been recently conducted to investigate the therapeutic effects of melatonin in PD patients. Melatonin was found to significantly improve sleep quality, non-motor symptoms and *Bmal1* expression. As previously mentioned, melatonin may be neuroprotective in alpha-synuclein pathology. However, recent studies found that melatonin was ineffective in eliminating insomnia and excessive daytime sleepiness [98,99]. Videnovic emphasises that practising proper sleep hygiene among all ages is a fundamental preventative factor to maintain healthy aging [100]. There is also potential for improved sleep hygiene practices as an ameliorative intervention in PD.

Another avenue for a therapeutic approach is treating prodromal disorders and comorbidities of PD. For instance, bright light therapy has been used in RCT studies and has been found to significantly improve insomnia, sleep quality, iRBD and depression in PD patients [100,101,102]. This approach also has the potential to address the underlying pathology by improving glymphatic clearance. Recognising these disorders may also provide opportunity for early diagnosis of PD or prevention. Finally, advancements in non-invasive brain imaging techniques such as diffusion tensor imaging in humans would be valuable in expanding current knowledge of glymphatic system pathology in PD as many studies are reliant on animal models to study interstitial clearance [14].

## 8. Conclusions

There is complex aetiology surrounding sleep and its disease-modifying role in PD pathogenesis and progression; causal links are yet to be established by human studies. Thus, AQP4 knockout in experimental PD resulted in the failure to increase TGF-β1 production which may exacerbate neuroinflammation [103]. Whereas in the atypical PD, multiple system atrophy, reduced perivascular AQP4 expression was associated with alpha-synuclein deposits and astrocyte activation [90]. Current evidence demonstrates that a bidirectional relationship exists between sleep disturbance and dopaminergic neurodegeneration. There are many pathological factors involved such as alpha-synuclein accumulation, neuroinflammation, glymphatic system impairment and circadian rhythm disruption. Sleep disturbance is a strong contributor to glymphatic dysfunction, which leads to diminished removal of alpha-synuclein. Thus, alpha-synuclein accumulates in the parenchyma to pathological levels with repeated sleep disruption. Conversely, alpha-synuclein has been shown to cause aggressive neuroinflammatory insults and AQP4 deficiency which perpetuates glymphatic impairment, causing dopaminergic degeneration in the SN as well as in other brain regions. Although a recent genetic study has shown associations between different AQP4 polymorphisms and varying rate of cognitive decline in PD [104], further work is vital to determine if a strong link exists between glymphatic dysfunction and the pathogenesis of the human disease.

## Figures and Tables

**Table 1:** Links between PD, sleep and glymphatic function.

Bidirectional Relationships	Article	Description
**Glymphatic system dysfunction in PD**	Chen and colleagues [50]	Basal ganglia perivascular spaces were associated with cognitive decline in PD patients at a 3-year follow up. BG-PVS and toxic protein levels in the CSF were significantly predictive of cognitive decline at 3-years.
	Chung and colleagues [51]	PD patients with enlarged basal ganglia perivascular spaces were older, had greater small vessel disease burden, more severely decreased dopamine transporter availability and higher freezing of gait risk compared to PD patients without enlarged PVS.
	Cui and colleagues [33]	Decreased aquaporin-4 expression in mice leads to further alpha-synuclein accumulation, dopaminergic neurodegeneration, impaired motor ability and glymphatic transport.
	Ma and colleagues [54]	Late-stage PD patients had significantly lower ALPS scores than normal controls.
	McKnight and colleagues [55]	ALPS-index was more reduced in PD patients compared to essential tremor patients. ALPS-index decreased with age.
	Li and colleagues [53]	Compared to controls, PD patients had more dilated perivascular spaces, greater expression of tau and decreased dopamine transporter binding.
	Park and colleagues [56]	PD patients experienced higher severity of basal ganglia perivascular spaces, white matter hypersensitivity, higher levodopa-equivalent dose, hypertension, and low mini-mental state examination scores. These factors were independent positive predictors of cognitive decline.
	Zou and colleagues [32]	Glymphatic CSF influx was reduced in A53T mice, and this effect was also accompanied by alpha-synuclein aggregation and AQP4 depolarisation. Disruption of meningeal lymphatic flow exacerbated alpha-synuclein pathology as well as induced neuroinflammation and dopaminergic neurodegeneration.
	Iliff and colleagues [34]	There is perivascular CSF influx, ISF efflux and clearance throughout the brain. Aquaporin-4 channels facilitate fluid clearance in the parenchyma as well as interstitial amyloid-beta clearance.
**Role of sleep in Glymphatic clearance**	Xie and colleagues [59]	Natural sleep or anaesthesia was associated with a 60% increase in interstitial space volume which contributed to a significant increase in convective CSF-ISF exchange and amyloid-beta clearance.
**Sleep disturbance and PD pathology**	Si and colleagues [79]	REM sleep behaviour disorder patients had a higher PVS burden than PD patients.
	Lysen and colleagues [73]	Worsening sleep quality and reduction in sleep duration were found to be associated with increased PD risk in adults in the next 6 years.
	Hsiao and colleagues [72]	Non-apnoea sleep disorders, particularly chronic insomnia, are associated with a higher risk of developing PD.
	Sohail and colleagues [74]	Increased sleep fragmentation was associated with Lewy body pathology, substantia nigra neurodegeneration and PD diagnosis risk.
	Breen and colleagues [67]	PD patients experienced increased sleep latency, reduced sleep efficiency, REM sleep and melatonin levels, elevated cortisol and abnormal *Bmal1* expression compared to controls.

**Abbreviations:** A53T—A53T alpha-synuclein mutation; ALPS—Analysis Along the Perivascular Space; AQP4—aquaporin 4; BG-PVS—basal ganglia perivascular space; CSF—cerebrospinal fluid; ISF—interstitial fluid; PD—Parkinson’s Disease; PVS—perivascular space; REM—rapid eye movement.
